# Supplementary material for: Effects of Rare Earth Elements on Blood Pressure and Their Exposure Biomarkers: Evidence from Animal Experiments
Source: Int J Environ Res Public Health. 2021 Sep 18;18(18):9836. doi: 10.3390/ijerph18189836 (PMC8469411; doi:10.3390/ijerph18189836)
Supplement: Supplementary file 1 [file ijerph-18-09836-s001.zip › ijerph-1354386-supplementary.pdf]

International Journal of
Environmental Research and Public Health

MDPI

Supplementary Materials

Table S1. The administrated doses of 14 rare earth element to rats.

No.	Full name	Abbr.	Rats' dose levels (mg [element]/(kg×day))		
			Low	Middle	High
1	Lanthanum	La	139	1387	13875
2	Cerium	Ce	114	1145	11445
3	Praseodymium	Pr	21.2	212	2117
4	Neodymium	Nd	73.2	732	7318
5	Samarium	Sm	16.5	165	1647
6	Europium	Eu	3.37	33.7	337
7	Gadolinium	Gd	13.4	134	1337
8	Terbium	Tb	2.51	25.1	251
9	Dysprosium	Dy	12.0	120	1199
10	Holmium	Ho	1.57	15.7	157
11	Erbium	Er	4.56	45.6	456
12	Thulium	Tm	0.85	8.48	84.8
13	Ytterbium	Yb	10.9	109	1093
14	Yttrium	Y	22.6	226	2262

Table S2. Comparison between the measured and referred hair concentrations (ng/g hair) of rare earth elements (REEs) of standard hair sample.

REEs	Measured	Referred
Lanthanum (La)	11.96 ± 0.33	13.4 ± 1.8 [a]
Cerium (Ce)	20.93 ± 1.2	19.7 ± 2.6 [a]
Praseodymium (Pr)	2.47 ± 0.17	(2.40) [b]
Neodymium (Nd)	8.65 ± 0.18	8.4 ± 1.5 [a]
Samarium (Sm)	1.78 ± 0.19	1.4 ± 0.4 [a]
Europium (Eu)	1.81 ± 0.11	(0.60) [b]
Gadolinium (Gd)	1.85 ± 0.21	(1.80) [b]
Terbium (Tb)	0.25 ± 0.02	(0.20) [b]
Dysprosium (Dy)	1.17 ± 0.14	(1.30) [b]
Holmium (Ho)	0.25 ± 0.04	(0.30) [b]
Erbium (Er)	0.70 ± 0.03	(0.70) [b]
Thulium (Tm)	0.09 ± 0.02	(0.10) [b]
Ytterbium (Yb)	0.59 ± 0.06	(0.80) [b]
Yttrium (Y)	7.44 ± 0.03	(7.50) [b]

[a] The concentration is provided by the National Standard Material Center in China with high accuracy; [b] The concentration is provided by the National Standard Material Center in China with relatively low accuracy.

Table S3. The limit of detection (LOD) and limit of quantification (LOQ) of REEs detections.

REEs	LOD (pg/mL)	LOQ (pg/mL)
Lanthanum (La)	<0.1	<10
Cerium (Ce)	<0.1	<10
Praseodymium (Pr)	<0.1	<10
Neodymium (Nd)	<0.1	<10
Samarium (Sm)	<0.1	<10
Europium (Eu)	<0.1	<10
Gadolinium (Gd)	<0.1	<10
Terbium (Tb)	<0.1	<10
Dysprosium (Dy)	<0.1	<10
Holmium (Ho)	<0.1	<10
Erbium (Er)	<0.1	<10
Thulium (Tm)	<0.1	<10
Ytterbium (Yb)	<0.1	<10
Yttrium (Y)	<0.1	<10

Table S4. The body weight change during the administration period.

Group	Administration Week											
	1	2	3	4	5	6	7	8	9	10	11	12
	Mean											
Blank	230 [a]	244	253	260	255	271	279	277	285	293	293	297
Low	235	256	260	267	279	282	279	288	292	295	298	302
Middle	234	251	267	280	285	297	297	289	300	306	310	310
High	236	248	257	258	264	281	289	288	299	301	303	303
	Standard deviation											
Blank	13	16	21	28	42	41	37	36	35	32	32	28
Low	12	14	16	19	24	30	32	28	29	30	29	33
Middle	10	11	14	16	22	20	9	24	18	17	17	10
High	15	20	15	20	16	9	12	17	13	11	18	17

[a] Body weight, unit: g.
